# Supplementary material for: BEX1 is a critical determinant of viral myocarditis
Source: PLoS Pathog. 2022 Feb 22;18(2):e1010342. doi: 10.1371/journal.ppat.1010342 (PMC8896894; doi:10.1371/journal.ppat.1010342)
Supplement: S1 Fig — A) Cytokine array representative blots where each membrane was incubated with four biological heart protein extract replicates from the indicated genotypes and treatments. B-V) Quantification of the cytokines observed in the heart of WT and BEX1 KO throughout the time course of CVB infection. (PDF) [file ppat.1010342.s001.pdf]

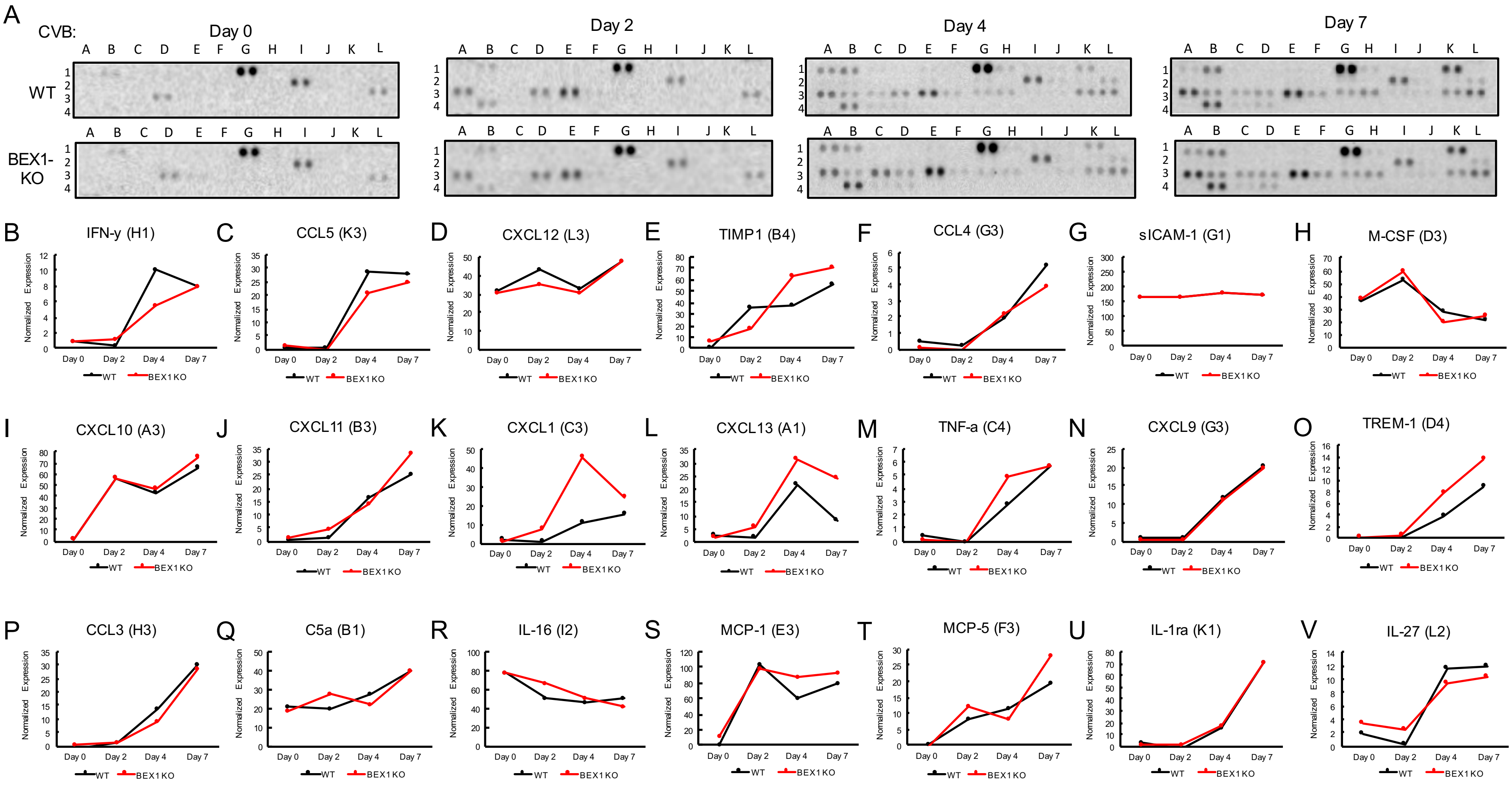

Supplemental Figure 1: Cytokine expression in WT and BEX1 KO hearts during CVB3 infection. A) Cytokine array representative blots where each membrane was incubated with four biological heart protein extract replicates from the indicated genotypes and treatments. B-V) Quantification of the cytokines observed in the heart of WT and BEX1 KO throughout the time course of CVB infection.
