# Supplementary material for: BEX1 is a critical determinant of viral myocarditis
Source: PLoS Pathog. 2022 Feb 22;18(2):e1010342. doi: 10.1371/journal.ppat.1010342 (PMC8896894; doi:10.1371/journal.ppat.1010342)
Supplement: S2 Fig — Effects of BEX1 overexpression on CVB-induced cardiac pathology: A-C) WT and BEX1-TG mice were compared in terms of their relative heart weight (A), percent ejection fraction (B), and cardiac fibrosis (C) in uninfected conditions and following 28 days of CVB infection. E-J) Flow cytometry showing the abundance of CD45+ total leukocytes (E), CD11b+ myeloid-derived cells (F), CD11b+/F4/80+ macrophages (G), CD11b+/LY6G+ neutrophils (H), CD11b-/CD3+ T lymphocytes (I), and CD19+ B lymphocytes (J) in the hearts of WT and BEX1-TG mice in uninfected conditions and following seven days of infection. (Error bars represent SEM; * p<0.05 CVB-treated WT vs. uninfected WT; & p<0.05 CVB-treated BEX1-TG vs. uninfected BEX1-TG; # p<0.05 BEX1-TG vs. WT same treatment). (PDF) [file ppat.1010342.s002.pdf]

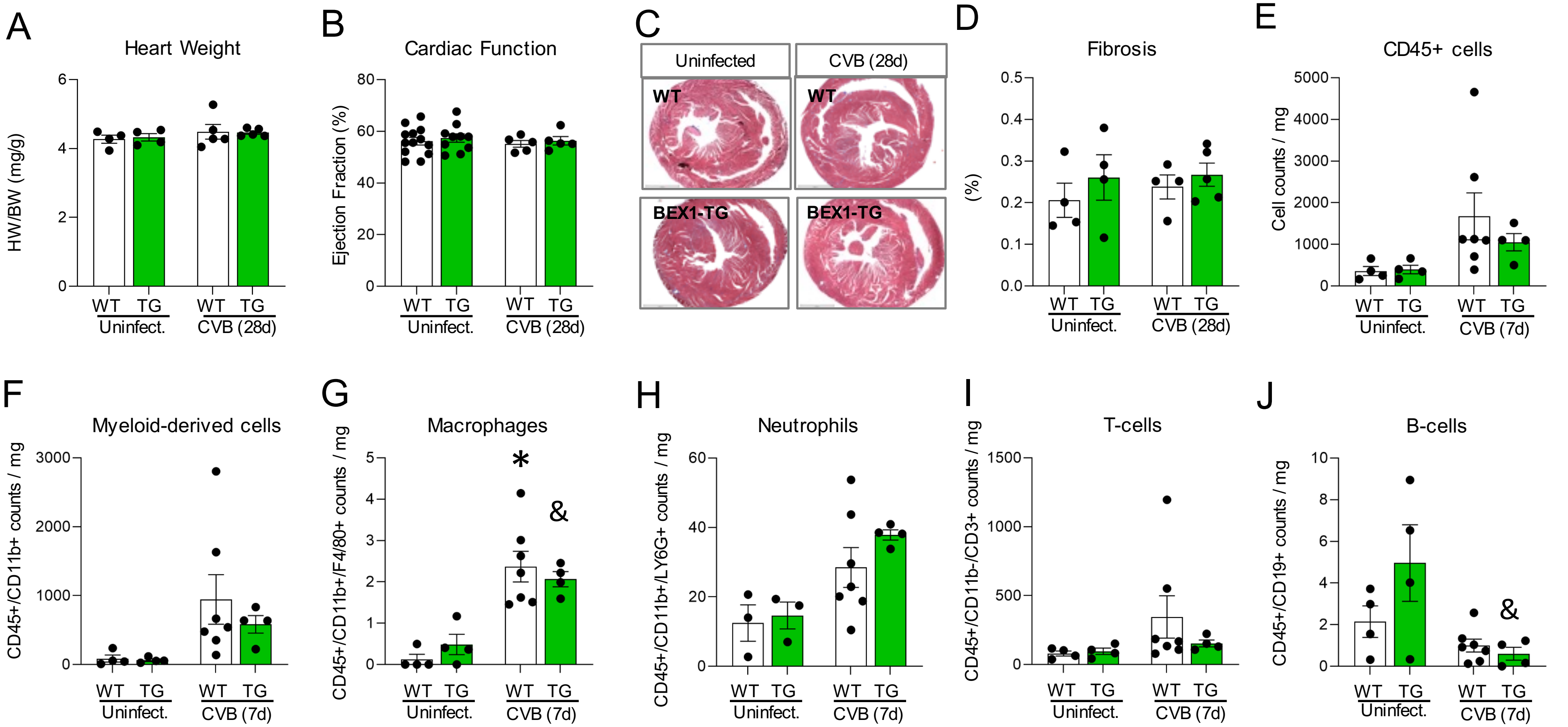

Supplemental Figure 2: Effects of BEX1 overexpression on CVB-induced cardiac pathology: A-C) WT and BEX1-TG mice were compared in terms of their relative heart weight (A), percent ejection fraction (B), and cardiac fibrosis (C) in uninfected conditions and following 28 days of CVB infection. E-J) Flow cytometry showing the abundance of CD45+ total leukocytes (E), CD11b+ myeloid-derived cells (F), CD11b+/F4/80+ macrophages (G), CD11b+/LY6G+ neutrophils (H), CD11b-/CD3+ T lymphocytes (I), and CD19+ B lymphocytes (J) in the hearts of WT and BEX1-TG mice in uninfected conditions and following seven days of infection. (Error bars represent SEM; * $p<0.05$ CVB-treated WT vs. uninfected WT; $^{\&}$ $p<0.05$ CVB-treated BEX1-TG vs. uninfected BEX1-TG; $^{\#}$ $p<0.05$ BEX1-TG vs. WT same treatment)
