# Supplementary material for: BEX1 is a critical determinant of viral myocarditis
Source: PLoS Pathog. 2022 Feb 22;18(2):e1010342. doi: 10.1371/journal.ppat.1010342 (PMC8896894; doi:10.1371/journal.ppat.1010342)
Supplement: S1 Table — IVS;d = interventricular septum thickness; diastole. IVS;s = interventricular septum thickness; systole. LVID;d = left ventricular internal diameter; diastole. LVID;s = left ventricular internal diameter; systole. LVPW;d = left ventricular posterior wall thickness; diastole. LVPW;s = left ventricular posterior wall thickness; systole. EF = ejection fraction. FS = fractional shortening. LV Vol;d = left ventricular volume; diastole. LV Vol;s = left ventricular volume; systole. SV = stroke volume. HR = heart rate. (# = p<0.05 WT Day 28 vs. BEX1-KO Day 28; * = p<0.05 WT Day 28 vs. WT Uninfected; & = p<0.05 BEX1-KO Day 28 vs. BEX1-KO Uninfected. (PDF) [file ppat.1010342.s003.pdf]

Supplemental Table 1: Echocardiographic measurements in uninfected and infected WT and BEX1-KO mice.

Parameter	WT Uninfected	BEX1-KO Uninfected	WT 28 Day CVB	BEX1-KO 28 Day CVB
**IVS;d**	0.913 (± 0.060)	0.989 (± 0.060)	0.814 (± 0.039)	0.646 (± 0.080) &
**IVS;s**	1.363 (± 0.074)	1.490 (± 0.027)	1.199 (± 0.028)	0.878 (± 0.121) # &
**LVID;d**	3.886 (± 0.063)	3.651 (± 0.108)	3.612 (± 0.098)	3.796 (± 0.172)
**LVID;s**	2.612 (± 0.074)	2.466 (± 0.090)	2.598 (± 0.046)	2.998 (± 0.171) # &
**LVPW;d**	0.742 (± 0.026)	0.786 (± 0.108)	0.853 (±0.083)	0.756 (± 0.086)
**LVPW;s**	1.052 (± 0.059)	1.014 (± 0.092)	1.092 (± 0.118)	0.896 (± 0.100)
**EF**	61.753 (± 2.216)	61.599 (± 1.952)	55.079 (± 1.167)	43.545 (± 2.676) # &
**FS**	32.778 (± 1.638)	32.473 (± 1.388)	27.993 (± 0.809)	21.119 (± 1.476) # &
**LV Vol;d**	65.452 (± 2.527)	56.620 (± 3.959)	55.134 (± 3.623)	62.396 (± 6.529)
**LV Vol;s**	25.021 (± 1.778)	21.774 (± 1.963)	24.621 (± 1.086)	35.511 (± 4.999) # &
**SV**	40.432 (± 2.243)	34.846 (± 2.669)	30.513 (± 2.594)*	26.885 (± 2.384)
**HR**	396.000 (± 27.528)	419.600 (± 26.292)	384.000 (± 21.959)	379.750 (± 18.870)

Supplemental Table 1: Echocardiographic measurements in uninfected and infected WT and BEX1-KO mice (mean ± standard error). IVS;d = interventricular septum thickness; diastole. IVS;s = interventricular septum thickness; systole. LVID;d = left ventricular internal diameter; diastole. LVID;s = left ventricular internal diameter; systole. LVPW;d = left ventricular posterior wall thickness; diastole. LVPW;s = left ventricular posterior wall thickness; systole. EF = ejection fraction. FS = fractional shortening. LV Vol;d = left ventricular volume; diastole. LV Vol;s = left ventricular volume; systole. SV = stroke volume. HR = heart rate. (# = $p<0.05$ WT Day 28 vs. BEX1-KO Day 28; * = $p<0.05$ WT Day 28 vs. WT Uninfected; & = $p<0.05$ BEX1-KO Day 28 vs. BEX1-KO Uninfected.
