# Supplementary material for: BEX1 is a critical determinant of viral myocarditis
Source: PLoS Pathog. 2022 Feb 22;18(2):e1010342. doi: 10.1371/journal.ppat.1010342 (PMC8896894; doi:10.1371/journal.ppat.1010342)
Supplement: S2 Table — IVS;d = interventricular septum thickness; diastole. IVS;s = interventricular septum thickness; systole. LVID;d = left ventricular internal diameter; diastole. LVID;s = left ventricular internal diameter; systole. LVPW;d = left ventricular posterior wall thickness; diastole. LVPW;s = left ventricular posterior wall thickness; systole. EF = ejection fraction. FS = fractional shortening. LV Vol;d = left ventricular volume; diastole. LV Vol;s = left ventricular volume; systole. SV = stroke volume. HR = heart rate. (# = p<0.05 WT Day 28 vs. BEX1-TG Day 28; * = p<0.05 WT Day 28 vs. WT Uninfected; & = p<0.05 BEX1-TG Day 28 vs. BEX1-TG Uninfected. (PDF) [file ppat.1010342.s004.pdf]

Supplemental Table 2: Echocardiographic measurements in uninfected and infected WT and BEX1-TG mice.

Parameter	WT Uninfected	BEX1-TG Uninfected	WT 28 Day CVB	BEX1-TG 28 Day CVB
**IVS;d**	0.939 (± 0.038)	0.903 (± 0.042)	0.798 (± 0.081)	0.777 (± 0.078)
**IVS;s**	1.348 (± 0.042)	1.376 (± 0.045)	1.194 (± 0.056)	1.156 (± 0.067) &
**LVID;d**	3.891 (± 0.062)	3.819 (± 0.059)	3.775 (± 0.055)	3.773 (± 0.111)
**LVID;s**	2.770 (± 0.076)	2.685 (± 0.061)	2.713 (± 0.056)	2.680 (± 0.074)
**LVPW;d**	0.673 (± 0.032)	0.786 (± 0.050)	0.701 (± 0.044)	0.791 (± 0.092)
**LVPW;s**	0.966 (± 0.029)	1.014 (± 0.050)	0.900 (± 0.068)	0.977 (± 0.055)
**EF**	56.163 (± 1.490)	57.382 (± 1.643)	55.144 (± 1.313)	56.320 (± 1.636)
**FS**	28.954 (± 0.964)	29.723 (± 1.119)	28.141 (± 0.843)	28.929 (± 1.129)
**LV Vol;d**	65.864 (± 2.522)	62.920 (± 2.355)	61.076 (± 2.117)	61.243 (± 4.171)
**LV Vol;s**	29.214 (± 2.033)	26.851 (± 1.466)	27.427 (± 1.400)	26.651 (± 1.823)
**SV**	36.650 (± 0.896)	36.070 (± 1.666)	33.649 (± 1.212)	34.591 (± 2.772)
**HR**	522.692 (± 21.742)	495.600 (± 19.602)	397.800 (± 29.847)*	435.000 (± 25.108)

Supplemental Table 2: Echocardiographic measurements in uninfected and infected WT and BEX1-TG mice (mean ± standard error). IVS;d = interventricular septum thickness; diastole. IVS;s = interventricular septum thickness; systole. LVID;d = left ventricular internal diameter; diastole. LVID;s = left ventricular internal diameter; systole. LVPW;d = left ventricular posterior wall thickness; diastole. LVPW;s = left ventricular posterior wall thickness; systole. EF = ejection fraction. FS = fractional shortening. LV Vol;d = left ventricular volume; diastole. LV Vol;s = left ventricular volume; systole. SV = stroke volume. HR = heart rate. (# = $p<0.05$ WT Day 28 vs. BEX1-TG Day 28; * = $p<0.05$ WT Day 28 vs. WT Uninfected; & = $p<0.05$ BEX1-TG Day 28 vs. BEX1-TG Uninfected.
