# Supplementary material for: BEX1 is a critical determinant of viral myocarditis
Source: PLoS Pathog. 2022 Feb 22;18(2):e1010342. doi: 10.1371/journal.ppat.1010342 (PMC8896894; doi:10.1371/journal.ppat.1010342)
Supplement: Attachment — Submitted filename: Answer to Reviewers_BEX1 and CVB_12.15.21.pdf [file ppat.1010342.s005.pdf]

Reviewer #1*: The authors of this study have demonstrated that BEX1 (Brain Expressed X-linked protein 1) is reduced in mice inoculated with coxsackievirus B3 (CVB3). BEX1 knockout (KO) mice have increased expression of CVB3 RNA in the heart as well as increased numbers of infected cardiomyocytes by day 7 implying that BEX1 expression limits the replication of these viruses in murine cardiomyocytes. In addition, the knockout mice have decreased immune cell infiltration at day 7 (normally the end of the peak of CVB3 replication in murine models of myocarditis) suggesting that BEX1 plays a role in increasing the cell mediated immune response. As might be expected, the BEX1 KO mice have decreased cardiac function and increased fibrosis at day 28 (the peak of peak of cardiac disease in the murine models of chronic myocarditis). This is interesting as it implies that the decreased inflammatory infiltration has resulted in increased damage from increased (perhaps persisting) replication of the virus due to less effective immune control of CVB3 infection. It is possible that one of the cytokines shown to be increased in the BEX1 KO mice during CVB3 infection has had effects leading to the loss of cardiac function but most of these do not persist to day 7 while the persistence of CVB3 in the heart to day 28 has been shown to correlate with susceptibility to late stage disease in the murine models of enterovirus-induced myocarditis (Chapman and Kim 2008 Curr Top Microbiol Immunol 323:275; Kandolf et al. 1999 Virus Res 62:149) as well as in human cardiomyopathy (Bouin et al. 2019 Circulation 139: 2326). It is a deficit that this study did not examine whether BEX1 is a factor in limiting the ability of the enterovirus CVB3 to persist to day 28 in the murine heart. These earlier studies in CVB3-induced myocarditis suggest that the antiviral effects of CVB3 may be more critical than the ability of BEX1 to induce infiltration of immune cells.*

The earlier publication by these authors (Accornero et al. 2017) in which they demonstrated that cardiac hypertrophy and cardiac functional loss due to transverse aortic constriction could be decreased in mice by a knockout of BEX1 or increased with overexpression of BEX1 suggested that BEX1 plays a role in increasing inflammation in response to pressure stress. The present study indicates that even with knockout of BEX, a process is occurring which produces cardiac disease in these infected mice. If the infiltration of immune cells is decreased even with higher levels of CVB3 at day 7, the effect of increased fibrosis and loss of function by day 28 are likely to be mediated directly by the virus. The authors also demonstrate that in murine embryonic fibroblast cultures, knockout of MEF increases replication of influenza and Sendai virus as well as CVB3, suggesting that the effect upon replication of CVB3 can be extended to other RNA viruses. That it can occur in the absence of an adaptive immune response is indicated by the decreased CVB3 replication in isolated cardiomyocytes from transgenic mice overexpressing BEX1. As the authors' earlier work indicated that BEX1 may have a role in RNP complexes shuttling and stabilizing mRNAs to be translated, I would suggest BEX1 might alter the ability of the innate response to mount an antiviral effect within the cell or might affect the ability of the virus to adapt the cellular environment of the cardiomyocyte to viral translation and replication. Examination of the persistence of CVB3 in day 28 hearts with knockout or overexpression of BEX1 would be valuable in this regard. In the absence of this data, the strong indications that the effects of BEX1 upon replication of CVB3 in cardiomyocytes is more likely to underly the ability of BEX1 to decrease CVB3 induced myocarditis and heart function loss should be discussed. I disagree with the statement that "Altogether we discovered BEX1 as a

novel determinant of cardiac viral infection that limits viral replication by regulating pro-inflammatory signals and immune cell recruitment."

We thank this reviewer for their work in assessing our manuscript and help in improving it. We completely agree with the raised points, in response to which we performed new experimentation as well as remodeled the manuscript and rephrased some of the conclusion statements as follows. In particular:

1. We have assessed the persistence of CVB in the heart of BEX1-KO mice at day 28 post-infection. The result showed that at day 28 the virus had been cleared in both control and KO hearts (**new Figure 1B**). While this excludes the possibility of long-term viral persistence, because cardiomyocytes are post-mitotic and the heart unable to regenerate at the age point used to conduct the experiments, it is plausible that virus-induced damage to cardiomyocytes leads to irreversible pathologic cardiac remodeling and dysfunction.
2. With the above point in mind, we do agree with the reviewer that direct cell damage is likely to play a major role in the observed phenotype, even more so than the defective immune cell recruitment. Indeed, we performed additional experiment in BEX1-overexpressing mice and we found that the ability of their hearts to limit viral load was independent from inflammatory cell recruitment (**new Supplemental Figure 2E-J**). In light of this result, we rephrased our conclusions to mitigate the emphasis on immune cell recruitment and focus more on the cell autonomous effects that we report in both isolated cardiomyocytes and MEFs.
3. To further strengthen our cell culture results, we analyzed the expression of interferon beta in MEFs with and without BEX1. The transcripts for interferon beta contain AU-rich elements, the RNA signature that we had discovered as regulated by BEX1 in our Accornero et al. 2017 paper (PMID 29192139). Interferon beta is also of particular relevance because it is directly produced by infected cells and regulates their ability to fight the virus, even in simplified systems where immune cell recruitment is not a factor. Interestingly, we found that interferon beta expression is defective in BEX1-KO cells infected by not only CVB, but also in response to influenza and sendai viruses (**new Figure 5D-F**). Moreover, interferon supplementation had a significant impact in rescuing the defective ability of BEX1-KO cells to limit viral replication (**new Figure 5G-I**).
4. With the above-described new results, we have accordingly modified the manuscript's conclusions and discussion (please see the revised manuscript version with tracked changes for an overview of all the modified text).

We believe that thanks to the reviewers' comments we now present a stronger, significantly improved study. We are therefore grateful for the received help.

Reviewer #2*: This paper looks at the involvement of BEX1 in the immune response in the heart after coxsackievirus B3 infection. The authors find that the immune response is dysregulated in BEX1 knockout mice after infection with the virus. While the concept is novel and interesting and potentially important, there are concerns to address.*

Reviewer #2: Concerns
1) Based on the data presented it might be an overinterpretation to call bex1 a "novel anti-viral agent" (or that "that BEX1 is an antiviral regulator") The stops short of showing a direct mechanism whereby BEX1 acts as an antiviral directly (the effect on viral infection in the KO and TG mice could be due to a number of secondary mechanisms. i.e. what if BEX1 regulates some aspect of immune cell function rather than a direct mechanism where it binds some viral component in cardiac myocytes?

We agree with this reviewer's comment. We have now performed additional experiments to better define direct versus immune cell-mediated BEX1 effects. In particular, we show that the ability of BEX1 to limit viral replication in the heart is independent from immune cell numbers (**new Supplemental Figure 2E-J**). We also more deeply assess the mechanism underlying the direct ability of BEX1 to protect the infected cell by regulating interferon beta expression (**new Figure 5D-I**). Altogether, we believe our data now better support an antiviral role for BEX1. Nevertheless, we also tempered some of our statements to avoid over interpretations.

2) Do bex1 tg mice have better cardiac function 28d after infection in support of the bex1 ko mice being worse off (fig. 4 d-f)? All the authors did was look at viral load 7d post infection with the tg mice.

This is again an important point. We performed these analyses; however, as CVB does not induce sufficient damage in wild type mice to worsen their cardiac function, we are not positioned to show cardioprotection with BEX1 overexpression (**new Supplemental Figure 2B-D**). Nevertheless, we agree on the importance to show this data as we can at least exclude any detrimental effect in our transgenic mice.

3) How are we to know that BEX1 would be better than an anti-viral agent through immunostimulants and immunosupressors (stated in intro paragraph 3) that are currently used to treat viral myocarditis?

The reviewer is right that we are far from being able to answer that question. However, in this revised manuscript we have performed additional experiments that focus on the ability of BEX1 to control viral replication, even independently from immune cell recruitment (**new Figure 5D-I**). We have therefore accordingly modified our conclusions, and we now better discuss the current limitations in tackling the exact contribution of both direct and immune-mediated BEX1 roles as anti-viral agent (please see last two paragraphs of the discussion).

4) figure 2: the graphs show no error bars for these blots. If they were run on n=4, where statistics run? Furthermore, why are 3 of the cytokine array data points in the supplement?

In light of the reviewers' comments and new results presented in this revised study, we have now moved the cytokine array data into **new Supplemental Figure 1**. We now included all detected

cytokines in the same figure (we initially had three in the supplement to have enough space in the main figure to present better visible results on some of the most interesting proteins). We ran this analysis by pooling four biological replicates into the same membrane to present the average result.

> *5) Paragraph 3 of results: "Similar results were seen at day 7 for other immune cell types (Figure 3D-G Supplemental Figure 2B-C)." Fig 2B is NOT lower for bex1 ko at 7 days and 2c is barely lower. Fig 2F is lower for bex1 hearts at day 7 but is not referenced. Still, only 2 of the 18 factors tested were actually lower for bex1 hearts, a bit of an overstatement by the authors as to the implied effects.*

We again agree with this reviewer. We have modified the correspondent result description to more precisely highlight the result in each panel.

> *Reviewer #2: Minor points:*
> *Ref 11 is for cells in culture. Is there a better ref for the cardiomyocytes being directly infected and lysed in vivo, considering that is what is trying to be referenced here?*

We now include references PMID 1309927 and 7689428, which better describe our point.

We are very grateful for this reviewer's help in improving our study and we believe that by accordingly modifying this manuscript we now present a stronger story.

> ***Reviewer #3**: It remains to be clarified how Bex1-ko mice with enhanced viral RNA content in the heart have impaired recruitment of immune cells. This is indeed a surprising result, particularly regarding to fibrotic injury depicted at later stages around d28. The authors should re-define their staining panels to define inflammatory immune cells in injured heart tissues, since the majority of infiltrating cells are oy myeloid origin and known to be monocytes. However, the authors failed to gate for monocytes and did not include a marker for this population. It deserves further investigation what the nature of the rise of F4/80+ cells is supposed to be after 28 days. Do these cells originate from invading monocytes? Although this might be intuitive, this interpretation is opposed by the lower abundance of inflammatory cells in Bex1-ko mice.*

We thank this reviewer for their suggestions and comments that helped us improve this study. We have now included analysis for monocyte markers (**new Figure 2E and 2J**). This was interesting as while the result showed a significant decrease in total monocytes in the heart of BEX1-KO mice 7 days post CVB infection (**new Figure 2E**), the CCR2+ subpopulation of cells were equally induced by the virus in the two genotypes (**new Figure 2J**). This could help explain the equal presence of F4/80+ cells 28 days post infection (now **Figure 2K**). We are therefore thankful to the reviewer for suggesting this improvement.

The manuscript lacks any information on the putative molecular function of the Bex1. The authors demonstrate the antiviral capacity by demonstrating lower viral RNA context for three infection models (in vitro, MEF, Fig.5). However, since the function of Bex1 proteins is not defined at a molecular level, the authors have to provide further information on the physiological role of this protein. In principal, their results might be attributed to altered cell survival, proliferation rate or whatever. A more detailed work-up of the infectious cycle/replication for the viruses is required to draw any conclusion on an "antiviral" function of Bex1. The interpretation particulary regarding the current pandemic SARS-CoV2 is highly speculative and not substantiated by their data.

We have now added more work to new Figure 5. In 2017 we had published an initial study where we found that BEX1 regulates AU-rich element containing transcripts (PMID 29192139). Interferon genes belong to this category. Interferon beta can be produced by infected cells independent from the immune system. We therefore tested if BEX1 modulates interferon beta expression in MEFs infected by CVB, Influenza and Sendai viruses. We found defective interferon beta expression in BEX1-KO cells independent from the tested virus (**new Figure 5D-F**). We also found that interferon beta supplementation has a significant effect in rescuing the increased viral replication observed in the absence of BEX1 (**new Figure 5G-I**). To note, while the rescue was complete in the case of Influenza virus, Sendai virus was still present at significantly higher level in the BEX1-KO cells and the same trend was observed with CVB. With this in mind, while we do believe interferon beta plays an important mechanistic role in the BEX1-dependent antiviral effects, we cannot exclude the contribution of additional factors. We have also analyzed apoptosis in cardiac cross-sections post CVB infections. While a trend for more apoptosis was observed in BEX1-KO hearts, it did not reach significance (see below, for reviewer only). This assay has the limitation of not differentiating between cardiomyoctes and non-myocyte cells; it is therefore hard to interpret this data especially considering that the number of non-myocytes might differ between injured control and BEX1-KO hearts. For this reason we decided to leave this piece of data out of the paper. We also now tempered the speculation on SARS-CoV2 since we did not test this particular virus in our study (revised end of first discussion paragraph).

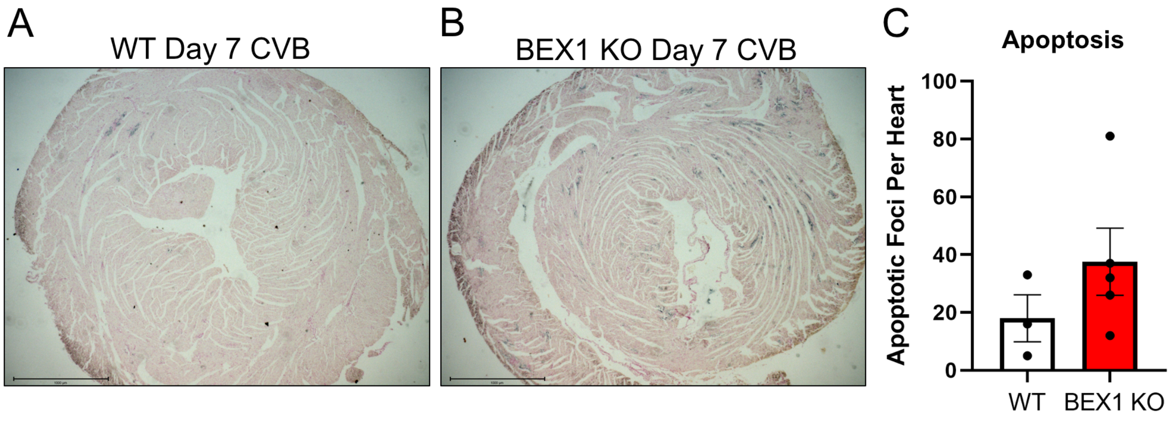

Apoptosis in CVB-infected mouse hearts. Apoptosis analysis carried out using CardioTACS in situ apoptosis detection kit (4827-30-K; R&D SYstems) on cardiac cross-sections from WT (A) and BEX1-KO (B) mice that were infected with CVB for 7 days. Apoptotic foci appear as dark blue. (C) Quantification of the number of apoptotic foci in the heart of the indicated mouse genotypes following 7 days of CVB infection using ImageJ.

The echocardiography data not to be shown in detail. Stroke volume, heart rate, Vol diastole, dimensions are the minimal requirements.

We have now included all echo parameters as **new Supplemental Table 1 and 2**.

The authors report on Bex1-dependent alterations in cytokine profiling. This reviewer has difficulties in appreciating a consistent regulation of cytokine expression. In Fig. 2J/K, chemokine responses are higher in BEX-1ko mice, which again is not reflected in the lower immune cell abundance presented in this strain in Fig. 3.

In agreement with this reviewer we have now moved the cytokine array result to supplemental data (**Supplemental Figure 1**). We agree that, although some interesting changes were observed, a clear elucidation of BEX1's role in inflammatory cytokine production is missing.

Information on the used CVB3 virus strain is required. 129Sv/Ev mice are quite resistant to CVB3 infection. With this genetic background it is mandatory to use littermate controls for all infections.

We now specify in the method section that we obtained the CVB3 strain from Jeffrey Bergelson from the University of Pennsylvania School of Medicine. We apologize for this missing information. We now also specify that we use littermate controls for all infections.

These data are missing for the transgenic mice: echo, flow cytometry heart tissue, cytokine response, histology.

We now added new transgenic mouse data as **new Supplemental Figure 2** and **new Supplemental Table 2**. To note, wild type mice were quite resistant to CVB-induced cardiomyopathy; consequently, we were not positioned to see protection in littermate transgenic mice. Based on also reviewers #1 and 2 comments this revised manuscript has reduced emphasis on the cytokine array (which was now moved to supplemental data); we therefore did not further analyze the cytokine response in the transgenic mice. Nevertheless, we agree that showing these additional echo, flow cytometry and histology data is informative in at least excluding any deleterious consequence of BEX1 overexpression in the heart.
